# Beneficial glycaemic effects of high-amylose barley bread compared to wheat bread in type 2 diabetes

**DOI:** 10.1038/s41430-023-01364-x

**Published:** 2023-11-08

**Authors:** Mette Bohl, Søren Gregersen, Yuyue Zhong, Kim Henrik Hebelstrup, Kjeld Hermansen

**Affiliations:** 1 Steno Diabetes Centre Aarhus, Aarhus University Hospital, 8200 Aarhus N, Denmark; 2 Diagnostic Centre, University Research Clinic for Innovative Patient Pathways, Silkeborg Regional Hospital, 8600 Silkeborg, Denmark; 3 Department of Clinical Medicine, Aarhus University, 8200 Aarhus N, Denmark; 4 Department of Plant and Environmental Sciences, University of Copenhagen, 1871 Frederiksberg C, Denmark; 5 Department of Agroecology, Section for Crop Genetics and Biotechnology, Aarhus University, 4200 Slagelse, Denmark; 6 Plantcarb Aps, 2970 Hørsholm, Denmark; 7 Department of Endocrinology and Internal Medicine, Aarhus University Hospital, 8200 Aarhus N, Denmark

**Keywords:** Type 2 diabetes, Diabetes

## Abstract

**Background:**

Cereals foods with a high content of dietary fibres or amylose have potential to lower postprandial glucose levels. Optimisation of cereal foods may improve management of type 2 diabetes (T2D).

**Methods:**

We investigated the impact on 4 h postprandial glucose responses given as incremental area under curve (iAUC) of bread made of either 50% RNAi-based (genetically modified) amylose-only barley flour (AmOn) (and 50% wheat flour), 50% hulless barley flour (and 50% wheat flour) or 75% hulless barley (and 25% wheat flour) in subjects with T2D compared with 100% wheat flour bread.

**Design:**

Twenty adults with T2D were randomly allocated to one of four breads at four separate visits. We measured fasting and 4 h postprandial responses of glucose, insulin, glucagon, triacylglycerol (TG), free fatty acids (FFA), glucagon-like peptide-1 (GLP-1) and gastric inhibitory polypeptide (GIP). Mixed model ANOVA was used to examine the differences.

**Results:**

Bread made from 50% AmOn lowered the 4 h postprandial glucose by 34%, 27%, 23% (*P* < 0.05) compared with 100% wheat, 50% or 75% hulless barley, respectively. Bread made from 75% hulless barley reduced the postprandial glucose response (iAUC) by 11% (*P* < 0.05) compared to 100% wheat bread.

Postprandial insulin responses (iAUC) were reduced for 50% AmOn compared with 100% wheat and 50% hulless barley and for 75% hulless compared to 50% hulless barley bread (*P* < 0.05). 4 h postprandial glucagon (tAUC) did not differ between the four bread types (*P* > 0.05). Lower postprandial GIP (iAUC) was observed after all barley breads compared to 100% wheat (*P* < 0.05), whereas no difference was seen in postprandial GLP-1. Postprandial TG and FFA (tAUC) were difficult to judge due to differences in fasting values.

**Conclusions:**

Bread made by replacing wheat flour with either 50% high-amylose or 75% hulless barley flour lowered postprandial glucose responses compared to 100% wheat bread indicating a beneficial impact on glucose regulation in T2D subjects.

This trial was registered at clinicaltrials.gov as NCT04646746.

## Introduction

The increasing prevalence of diabetes is primarily due to obesity, lack of physical activity and unhealthy diet. Therefore, it is important to identify and generate foods and diets that counteracts the development of type 2 diabetes (T2D). Diets low in glycaemic index (GI) and glycaemic load have the potential to prevent T2D [1–5] or to improve management of T2D and cardiovascular diseases [6].

Cereals e.g., rice, wheat, rye, oat and barley are staple foods worldwide. The content of dietary fibres [7–9] and the composition of starch both affect glucose metabolism in diabetic [10] and non-diabetic subjects [11, 12]. Starch consists of amylose (being mostly linear 1,4-α-glucan) and amylopectin (a branched polymer that mainly consists of 1,4-α linkages with branches of 1,6-α-glucan types). The amount of amylose is between 15-30% in most cereal and tuberous crops. In barley, three different *starch branching enzymes* (*SBE*s) are involved in the formation of amylopectin. By suppressing these three *SBE* genes, amylopectin formation is virtually blocked and only amylose and amylose-like glucans are formed [13]. By genetic modification, this formation was achieved in barley hereby increasing the amount of amylose to more than 99% of the starch [14]. In vitro this type of gene-modified high amylose barley (AmOn) suppressed GI to 10-15 [15].

High-amylose starches are more slowly degraded and contain a relatively higher amount of resistant starch (RS) compared to normal-amylose starches [13, 14]. Slowly digestible starch and higher amount of RS have shown to decreases postprandial glucose compared to low RS controls with same amount of total carbohydrate [16, 17].

Barley flour has lower GI in humans than wheat flour [18, 19] and possesses positive effects on the gut microbiome, endogenous gut hormones responses and appetite regulation [20]. Most cultivated barley varieties are hulled. In western countries barley contributes very little as staple food but is used for livestock feed and to produce beer malt. Interestingly, barley has a number of favourable properties that we can take advantages of. Farmers use significantly less nitrogen for barley fields than for wheat fields and barley tolerates climate changes better than wheat [21]. For bread making hulless barley is preferred over hulled barley due to better baking qualities [22], but it is not yet clarified how hulless barley affects GI in T2D. Nor has it been clarified in normal subjects or subjects with T2D how genetically modified high-amylose barley affects glucose metabolism. It should be underlined that not only glucose responses but also insulin, glucagon, incretins and lipid responses are abnormal in T2D and may play important metabolic roles in the pathogenesis.

The aim was to study the acute effects of breads of different contents of genetically modified high-amylose (AmOn) barley as well as hulless barley on glycaemic responses in subjects with T2D. Concomitantly, we measured acute changes in relevant hormones and lipid responses being abnormal in T2D. We hypothesised that bread made from AmOn or hulless barley improves postprandial glucose responses, given as incremental area under curve (iAUC), compared to wheat bread in subjects with T2D.

## Subjects and methods

### Study design

The study was performed as an acute randomised, single-blinded, intervention with four test meals consisting of breads made with a mix of hulless barley flour (50% or 75%) or AmOn flour (50%) with wheat flour (50% or 25%) and compared to 100% wheat flour bread. The study participants were randomised to the order of visits using RedCap®.

The primary outcome, postprandial glucose response, was evaluated by changes in the incremental area under the glucose response curve. The secondary outcomes were postprandial changes in insulin, glucagon, triglyceride (TG), free fatty acids (FFA), glucagon like peptide 1 (GLP-1), and gastric inhibitory polypeptide (GIP) responses given as either iAUC or total AUC (tAUC).

### Study participants

Twenty Caucasian subjects with T2D were recruited through local newspapers and electronic advertisement to participate in the study. The study was conducted at the Department of Endocrinology and Internal Medicine, Aarhus University Hospital, between March 2021 and October 2021. After receiving written and oral information, all subjects gave their written informed consent before participating in the study. Subjects were screened on the basis of their medical history and a physical examination. The inclusion criteria were as follows: Adults ≥18 years of age. Diabetes based on International Diabetes Federation criteria (https://apps.who.int/iris/bitstream/handle/10665/43588/9241594934_eng.pdf) with on-treatment haemoglobin A1c (HbA1c) between 42 and 78. All anti-diabetic medications were allowed except the below mentioned.

The exclusion criteria were: type 1 diabetes, insulin treated T2D, use of weekly administrated GLP-1 agonist, use of acarbose, significant cardiovascular, kidney, liver or endocrine comorbidity, significant psychiatric history, treatment with steroids, alcohol or drug abuse, pregnancy or breastfeeding, or legal incompetence.

Treatment with drugs for hypertension or high cholesterol was allowed if the treatment dose was stable throughout the study period.

The study protocol was carried out in accordance with the Helsinki Declaration of 1975 as revised in 1983 and was approved by the Central Denmark Region Committees on Health Research Ethics (1-10-72-299-20). The study was registered at clinicaltrials.gov as NCT04646746.

### Production of dietary supplements and dietary assessment

All participants were provided with a standard meal for dinner the night before each study day. The meals were commercially produced ‘chili con carne’ (Salling Group A/S, Denmark). The women’s meal contained 536 kJ and the men’s meal contained 638 kJ.

Bread with 100% wheat flour (standard commercial Manitoba wheat flour, HavneMøllen, Vejle, Denmark) and bread mixed of wheat and hulless barley flour were produced by ‘P.A. Andersen bakery’ (Vejle, Denmark), whereas bread made from a mixture of wheat and AmOn flour was produced at ‘Plantcarb ApS’ (Slagelse, Denmark), since genetically modified flour is not allowed in Danish commercial bakeries. The grains were grown in field plots over the summer of the year 2020 at Aarhus University (Flakkebjerg, Denmark). Whole grains of AmOn were ground into flour by a Komo Fidibus 21 benchtop mill (KOMO GMBH & CO. KG, Hopfgarten, Germany). The breeding and characterisation of starch from the AmOn barley variety has previously been described [14]. In brief, the AmOn was based on a hulled barley variant (*H. vulgare* var. golden promise) and was bred to contain more than 99% amylose of the starch fraction by introducing a chimeric RNAi hairpin DNA construct that simultaneously reduced the expression levels of the genes *SBEIIa*, *SBEIIb* and *SBEI* by more than 90%. The hulless barley (*H. vulgare* var. PS3) used in this study was developed by the breeder Agrologica (Mariager, Denmark).

Similar recipes were used for the four breads. The amount of total carbohydrate was calculated using ‘Vitakost ApS’ (Kolding, Denmark) and the amount of bread corresponding to 50 g of total carbohydrate was estimated. All four bread types were packed in individual sealed bags containing either 114 g (100% wheat), 119 g (50% hulless barley), 122 g (75% hulless barley) or 128 g (50% AmOn) of bread corresponding to 50 g of estimated total carbohydrate. Hereafter the bread was frozen at –20 °C. At the study days the bread was taken out of the freezer and defrosted without being heated.

### Bread component analysis

The moisture content was determined by the weight loss after drying in a 120 °C oven for 24 hours. The total carbohydrate content was measured as the sum of the fibre and starch content. The Megazyme K-TSTA kit (Megazyme, Co. Wicklow, Ireland) was used to measure the total starch content of samples containing resistant starch following the manufacturer’s instructions. This method variant uses dimethyl sulfoxide and boiling bath, and dissolution in dimethyl sulfoxide at 100 °C is effective to solubilize all starches in the bread, including the resistant starch. The fibre content was determined using the Megazyme total fibre assay kit (K-TDFR-200A, Megazyme, Co. Wicklow, Ireland) following the manufacturer’s instructions. The K-TDFR-200A kit measure mainly the cell wall polysaccharides and some RS.

### Visits

After signing the informed consent form, screening blood samples were analysed for alanine aminotransferase, HbA1c, fasting glucose, TG, thyroid-stimulating hormone, sodium, potassium, creatinine, and haemoglobin to rule out intercurrent disease and to ensure that the inclusion criteria of HbA1c was fulfilled.

After a standardised evening meal and an overnight fast (from midnight), the study participants arrived at the clinic between 07.00–07.30 AM at all four study days. A catheter was placed in a cubital vein for blood sampling. Baseline blood samples were drawn (at timepoints –10 and 0 min), and then the test bread was consumed within the next 10 min together with 250 ml of tap water.

During the following four hours blood samples were drawn at specified time points: glucose, insulin, and glucagon at –10, 0, 10, 20, 30, 45, 60, 90, 120, 150, 180, 210, and 240 min; TG, FFA, GLP-1 and GIP at –10, 0, 30, 60, 120, 180, and 240 min. All blood samples were immediately centrifuged at 3000 *g* for 10 min at 4 °C; thereafter, the plasma samples were frozen at –20 °C and the next day stored at –80 °C.

Smoking was not allowed during the overnight fast or during the study visits. Alcohol consumption was not permitted for two days before the fasting visits and strenuous exercise was not permitted the day before the fasting visits. Anti-hypertensive, cholesterol-lowering drugs and anti-diabetic drugs were paused 24 hours before every study day. The four intervention days were separated by at washout period of minimum six days.

### Blood analyses

Plasma glucose was measured by a glucose oxidase method with a GOD-PAP glucose kit (no. 11491253216; Roche Diagnostics GmbH, Germany). Serum insulin and glucagon were measured with ELISA (insulin no. 10-1113-01 and glucagon no. 10-1271-01; Mercodia AB, Sweden). Plasma TG and FFA concentrations were measured with enzymatic colorimetric assays by using commercial kits (No. 04657594190, Roche Diagnostics GmbH, Germany, for TG and code 270–7700, Wako Chemicals GmbH, Germany, for FFA). Measurements of both parameters were made on a Cobas c111 analyser (Roche Diagnostics GmbH, Germany). GLP-1 and GIP were measured with NL-ELISA techniques (GLP-1 no. 10-1278-01 and GIP no. 10-1258-01; Mercodia AB, Sweden).

### Statistical analysis

The power calculation was based on previous results from our group and made to detect a difference in our primary outcome (i.e., postprandial glucose response, given as iAUC) of 20% between diets [11]. The number of participants needed to complete the study and achieve a statistical power of 80% was calculated to be 20 (*a* < 0.05, *b* = 0.80). A mixed model ANOVA was used to examine the effect of each bread type compared with the wheat bread (control). *P* < 0.05 was considered statistically significant. Results are given as mean *±* 95% confidence interval (CI) in tables and as mean *±* SEM in graphs, unless otherwise stated. Fasting values of the outcomes are presented in Table 3, whereas postprandial changes, given as changes in percentages from fasting value, are presented in graphs along with the corresponding iAUC (area above fasting value) for glucose, insulin, GLP-1 and GIP, and as tAUC (area above zero) for glucagon, TG and FFA. All statistical calculations were performed with STATA version 17 (StataCorp LP, Texas, USA) and GraphPad Prism 6 (GraphPad Software, Boston, USA) was used to generate the graphical elements.

## Results

### Baseline clinical characteristics

20 participants were randomised and 18 completed all meal tests. One participant was excluded since no intravenous access could be obtained and one dropped out due to personal reasons. Table 1 presents baseline clinical characteristics of the 18 completing participants.

**Table 1:** Baseline characteristics of the 18 completing participants with type 2 diabetes (mean values ± SEMs; ranges in parentheses).

18 completing participants	
Gender (female, *n* (%))	13 (72%)
Age (y)	60.5 ± 2.7 (38-75)
HbA1c (mmol/mol)	49.7 ± 1.1 (43-59)
BMI (kg/m^2^)	30.5 ± 1.1 (19–44.8)
Total cholesterol (mmol/L)	4.1 ± 0.1 (3.1–4.9)
LDL-cholesterol (mmol/L)	2.0 ± 0.1 (1.2–1.7)
HDL-cholesterol (mmol/L)	1.2 ± 0.1 (1.0–1.7)
Statin use *n* (%)	13 (72%)
Anti-hypertensive drugs *n* (%)	12 (67%)
Metformin *n* (%)	15 (83%)
Other oral antidiabetics *n* (%)	5 (28%)
Smoking *n* (%)	3 (17%)

*HbA1c* haemoglobin A1c, *BMI* body mass index, *LDL* low-density lipoprotein, *HDL* high-density lipoprotein.

72% of the participants were female; average age was 61 years (±SEM: 2.7); average HbA1c was 50 mmol/mol (±SEM: 1.1); and average BMI was 31 kg/m^2^ (±SEM: 1.1).

### Bread fibre and starch content

Table 2 presents bread analyses for total carbohydrate, fibre and starch content in the four bread types given as both gram (g)/100 g and g/serving. Total carbohydrate is the sum of dietary fibre and total starch. The total carbohydrate content in g/100 g did not differ between groups (*P* > 0.05); however, due to our pre-study calculations the four test meals differed in g/serving. The study participants consumed 49.3 g/serving (SD ± 0.4), 54.4 g/serving (SD ± 0.6), 51.9 g/serving (SD ± 0.3) and 57.1 g/serving (SD ± 0.9) of total carbohydrate from the 100% wheat, 50% hulless barley, 75% hulless barley and 50% AmOn bread, respectively. Total fibre content differed between groups (*P* < 0.05) with 16.4 g/serving (SD ± 0.6), 11.2 g/serving (SD ± 0.3), 9.2 g/serving (SD ± 0.5) and 5 g/serving (SD ± 0.0) for 50% AmOn, 50% hulless barley, 75% hulless barley and 100% wheat bread, respectively. It cannot be ruled out, that a fraction of the hard to degrade amylose is accounted for in the total fibre results. Total starch content was similar in the 100% wheat and 50% hulless barley bread, with 44.3 g/serving (SD ± 0.5) and 45.1 g/serving (SD ± 0.5), respectively, (*P* > 0.05). The total starch content for 75% hulless barley and 50% AmOn was similar with 40.7 g/serving (SD ± 0.4) and 40.8 g/serving (SD ± 1.3), respectively, (*P* > 0.05).

**Table 2:** The moisture, total carbohydrate, total fiber and total starch content of bread samples given as g/100 g and g/serving in the table (mean values ± SD with three replicates).

Bread types	Moisture content (g/100 g)	Total carbohydrate (g/100 g)	Total carbohydrate (g/serving)*	Total fibre content (g/100 g)	Total fibre content (g/serving)	Total starch content (g/100 g)	Total starch content (g/serving)
100% wheat	44 ± 4^a^	43.3 ± 0.4^a^	49.3 ± 0.4^d^	4.4 ± 0.0^d^	5.0 ± 0.0^d^	38.9 ± 0.5^a^	44.3 ± 0.5^a^
50% hulless barley	41 ± 2^a^	45.7 ± 0.5^a^	54.4 ± 0.6^b^	7.8 ± 0.4^c^	9.2 ± 0.5^c^	37.9 ± 0.4^b^	45.1 ± 0.5^a^
75% hulless barley	45 ± 0^a^	42.5 ± 0.2^a^	51.9 ± 0.3^c^	9.2 ± 0.3^b^	11.2 ± 0.3^b^	33.3 ± 0.3^c^	40.7 ± 0.4^b^
50% AmOn	41 ± 6^a^	44.6 ± 0.7^a^	57.1 ± 0.9^a^	12.8 ± 0.5^a^	16.4 ± 0.6^a^	31.9 ± 1.0^d^	40.8 ± 1.3^b^

Values with different letters (a, b, c, d) in the same column are significantly different at *P* < 0.05. *Total carbohydrate is the sum of dietary fibre and total starch.

### Plasma glucose, insulin and glucagon responses

**Fig. 1:**
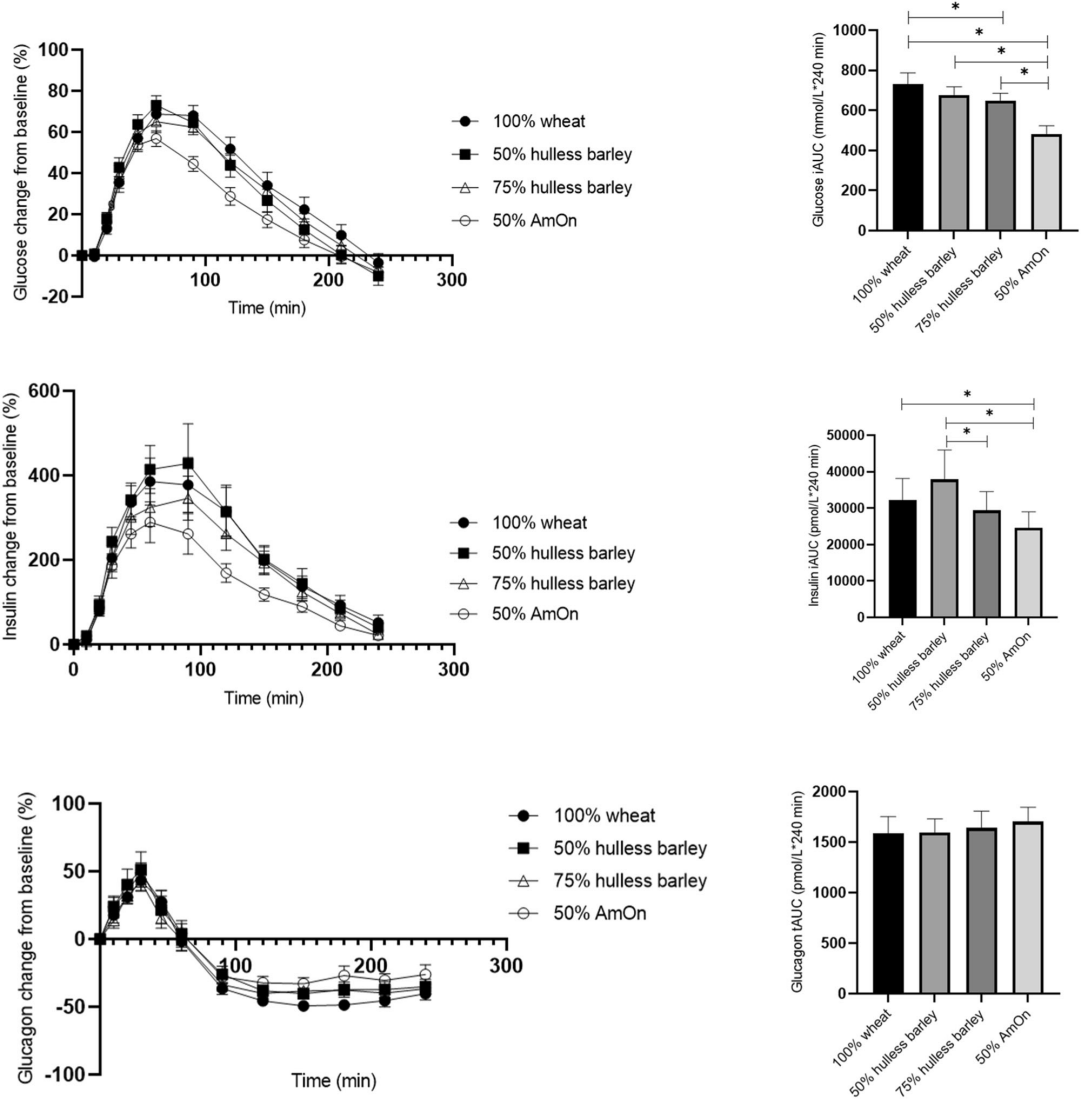
Left: Shows mean (±SEM) changes in percentage from baseline in plasma glucose, insulin and glucagon in 18 subjects with type 2 diabetes to a test meal of either 100% wheat flour bread (100% wheat), bread with a mix of 50% hulless barley flour and 50% wheat flour (50% hulless barley), bread mixed of 75% hulless barley flour and 25% wheat flour (75% hulless barley) or bread mixed of 50% amylose-only barley flour and 50% wheat flour (50% AmOn). Right: Shows mean (±SEM) incremental area under curve (iAUC) of glucose, and insulin and total area under curve (tAUC) for glucagon. *Significantly different from each other (*P* < 0.05).

Fasting plasma concentrations of glucose, insulin and glucagon are given in Table 3. No difference in fasting values was observed between test meals (*P* > 0.05). Mean percentage changes (±SEM) from baseline in glucose, insulin and glucagon are presented in Fig. 1 along with the corresponding iAUC for glucose and insulin and tAUC for glucagon.

**Table 3:** Baseline fasting values of plasma glucose, insulin, glucagon, gastric inhibitory polypeptide (GIP), glucagon like peptide-1 (GLP1), triglyceride (TG) and free fatty acids (FFA) (mean values with 95% CI in parentheses)^1^.

	100% wheat	50% hulless barley	75% hulless barley	50% AmOn
Glucose fasting (mmol/L), baseline	8.0 (7.5, 8.6)	7.8 (7.2, 8.3)	7.8 (7.3, 8.4)	7.6 (7.1, 8.2)
Insulin fasting (pmol/L), baseline	81.1 (54.0, 108.3)	71.6 (44.5, 98.8)	70.5 (43.3, 97.7)	76.1 (49.0, 103.3)
Glucagon fasting (pmol/L), baseline	9.2 (7.4, 11.0)	8.7 (6.9, 10.6)	8.9 (7.1, 10.7)	8.5 (6.7, 10.4)
GIP fasting (pmol/L), baseline	10.3 (0.9, 19.8)	20.5 (11.0, 30.0)	11.2 (1.8, 20.7)	11.6 (2.2, 21.1)
GLP1 fasting (pmol/L), baseline	8.3 (6.4, 10.1)	8.5 (6.6, 10.3)	8.1 (6.2, 9.9)	7.4 (5.6, 9.3)
TG fasting (mmol/L), baseline	1.5 (1.3, 1.7)^a^	1.4 (1.2, 1.6)^b^	1.6 (1.4, 1.8)^a,b^	1.5 (1.3, 1.7)
FFA fasting (mmol/L), fasting	0.8 (0.7, 0.9)^a^	0.7 (0.6, 0.8)^a^	0.8 (0.7, 0.9)	0.7 (0.6, 0.8)

^1^Values with similar letters (a, b, c, d) in same row differ from each other (*P* < 0.05).

Postprandial glucose responses (iAUCs) for 50% AmOn were reduced by 34% (248 mmol/L*240 min (95% CI: 175, 321; *P* < 0.001)), 27% (194 mmol/L*240 min (95% CI: 121, 267; *P* < 0.001)) and 23% (167 mmol/L*240 min) (95% CI: 93, 240; *P* < 0.001) compared with 100% wheat, 50% or 75% hulless barley flour bread, respectively.

Postprandial glucose response was reduced by 11% (81 mmol/L*240 min (95% CI: 8, 155; *P* = 0.030)) for bread made with 75% hulless barley compared with that of 100% wheat flour bread.

Glucose peaks compared to fasting glucose for bread made with 50% AmOn were 1.5 mmol/L (95% CI: 0.9, 2.0; *P* < 0.001), 1.4 mmol/L (95% CI; 0.9, 2.0; *P* < 0.001) and 1.0 mmol/L (95% CI; 0.4, 1.5; *P* = 0.001) lower than breads made with 100% wheat, 50% or 75% hulless barley, respectively.

Postprandial insulin responses (iAUCs) were reduced by 24% (7.7 nmol/L * 240 min (95% CI; 0.5, 14.9; *P* = 0.035)) and 35% (13.3 nmol/L * 240 min (95% CI: 6.2, 20.5; *P* < 0.001)) for 50% AmOn compared with 100% wheat and 50% hulless barley, respectively. Postprandial insulin did not differ between wheat and 50% hulless barley (*P* = 0.121) or between wheat and 75% hulless barley (*P* = 0.422). However, insulin was reduced by 22% (8.5 nmol/L * 240 min (95% CI; 1.3, 15.7; *P* = 0.021)) for 75% hulless barley compared with 50% hulless barley.

Postprandial glucagon responses (tAUCs) did not differ between groups (*P* > 0.05).

### GIP and GLP-1 responses

**Fig. 2:**
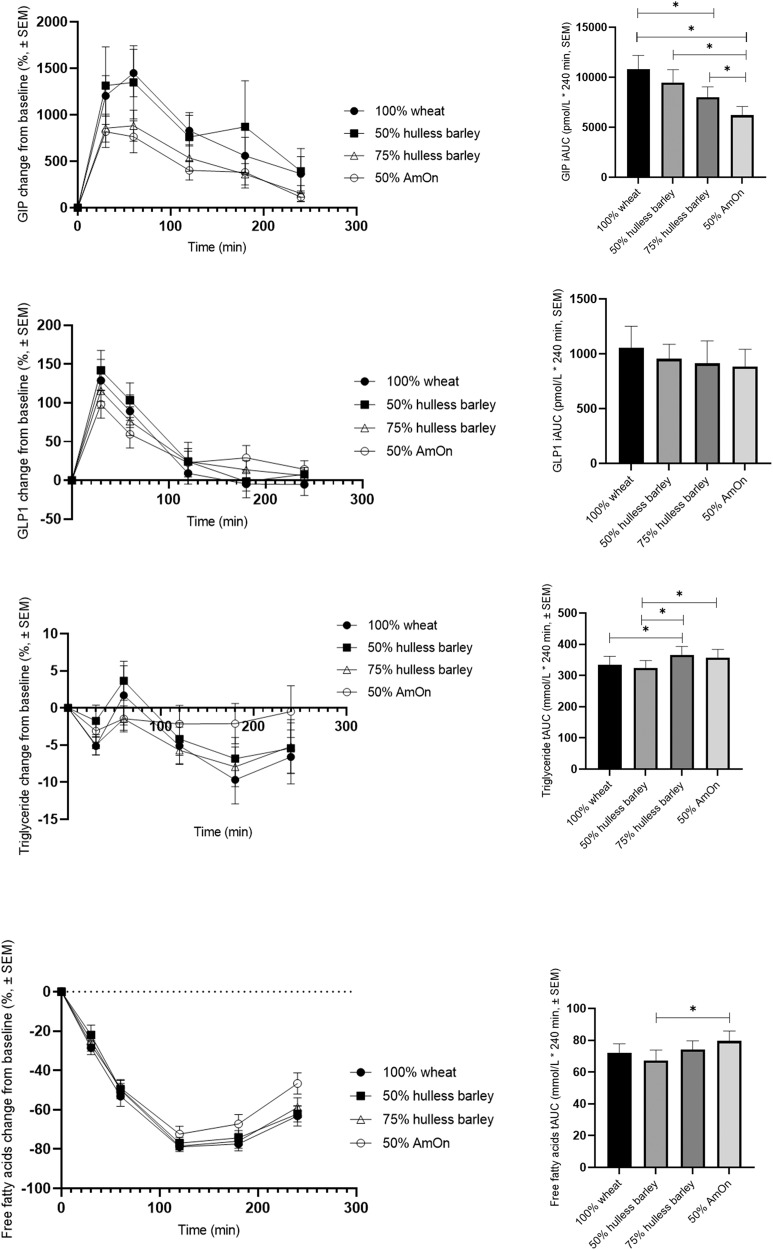
Left: Shows mean (±SEM) changes in percentage from baseline in plasma gastric inhibitory peptide (GIP), glucagon like peptide 1 (GLP-1), triglyceride (TG) and free fatty acids (FFA) in 18 subjects with type 2 diabetes to a test meal of either 100% wheat flour bread (100% wheat), bread with a mix of 50% hulless barley flour and 50% wheat flour (50% hulless barley), bread mixed of 75% hulless barley flour and 25% wheat flour (75% hulless barley) or bread mixed of 50% amylose-only barley flour and 50% wheat flour (50% AmOn). Right: Shows mean (±SEM) incremental area under curve (iAUC) of GIP and GLP-1 and total area under curve (tAUC) for TG and FFA. *Significantly different from each other (*P* < 0.05).

In Fig. 2 we present concentrations of GIP and GLP-1 as mean changes (±SEM) from baseline in percentage, together with the postprandial iAUC responses. Fasting values of GIP and GLP-1 are presented in Table 3.

Fasting GIP and GLP-1 did not differ between test meals (*P* > 0.05).

After intake of test meals based on AmOn flour iAUCs for GIP were reduced by 4.6 nmol/L * 240 min (95% CI: 3.0, 6.2; *P* < 0.001), 3.2 nmol/L * 240 min (95% CI: 1.6, 4.9; *P* < 0.001) and 1.8 nmol/L * 240 min (95% CI: 0.2, 3.4; *P* = 0.032) compared to 100% wheat, 50% hulless barley and 75% hulless barley, respectively. 75% hulless barley reduced the postprandial GIP response by 2.8 nmol/L * 240 min (95% CI: 1.2, 4.5; *P* = 0.001) compared with 100% wheat.

The postprandial GLP-1 responses did not differ among the four test meals (*P* > 0.05).

### FFA and TG responses

Mean fasting values of TG and FFA are given in Table 3. By chance fasting TG was increased with 75% hulless compared to both 50% hulless and 100% wheat bread and FFA was higher with 100% wheat than with 50% hulless barley (*P* < 0.05). This cannot be related to the individual bread type, due to the acute design; however, we cannot rule out that it might reflect the postprandial changes. Changes in concentrations of FFA and TG as mean changes (±SEM) from baseline in percentage together with the postprandial responses given as tAUC are presented in Fig. 2. After intake om 75% hulless barley the postprandial TG t responses (given as tAUC) was increased by 31.8 mmol/L * 240 min (95% CI: 3.6, 60.0; *P* = 0.028) and 41.3 mmol/L * 240 min (95% CI: 13.1, 69.6; *P* = 0.005) compared to 100% wheat and 50% hulless barley, respectively. Postprandial TG (tAUC) was reduced by 31.8 mmol/L * 240 min (95% CI: 3.6, 60.1, *P* = 0.028) after 50% hulless barley compared to 100% wheat. The postprandial FFA responses were suppressed after alle four diets, but more after 50% hulless barley than after 50% AmOn (tAUC) was reduced by 12.4 mmol/L * 240 min (95% CI: 4.6, 20.0; *P* = 0.002)

## Discussion

The present study evaluated acute effects of replacing wheat bread with either 50% of genetically modified high amylose barley flour (AmOn) or 50% or 75% hulless barley flour on postprandial glucose metabolism in subjects with T2D compared to 100% wheat flour bread.

Our major finding was that by replacing 50% of the wheat flour with 50% of the genetically modified high-amylose barley AmOn, postprandial glucose responses were reduced by 34%, 27%, 23% (*P* < 0.05) compared with 100% wheat flour bread, 50% hulless barley flour bread or 75% hulless barley flour bread, respectively, even though containing the most total carbohydrate of the four breads. Furthermore, by replacing 75% of wheat flour by hulless barley the postprandial glucose response was reduced by 11% compared to 100% wheat flour bread. In addition, the postprandial insulin response after 50% AmOn bread was lower than after 100% wheat bread and 50% hulless barley, indicating that the difference in glycaemic response was not caused by higher postprandial insulin levels. Interestingly, GIP responses were lower for AmOn bread than the three other breads, probably due to the higher fibre content and lower glucose response to AmOn bread [23]. Similar GLP-1 responses were detected to the four bread types. FFAs were suppressed after all four meal tests, however less after 50% AmOn compared to 50% hulless barley; however, this result is a bit difficult to judge, since fasting FFA was lower in the 50% hulless barley group compared with 100% wheat, but not compared to 50% AmOn. The difference in fasting values is by chance. The effect of the test meals on the postprandial TG responses is also difficult to judge, since there were significantly differences by change between groups at baseline. The baseline differences in lipids might have been overcome by standardizing the pre-study diet for a longer period.

Lowering the postprandial glycaemic responses in diabetes is important. Thus a diet low in GI and glycaemic load is optimal for prevention and management of T2D, cardiovascular diseases and mortality [5].

Despite similar content of total carbohydrate (g/100 g), the fibre content (both as g/100 g and g/serving) in 50% AmOn bread was increased compared with 100% wheat, 50% or 75% hulless barley bread, respectively. In addition, the total fibre content was increased in 50% and 75% hulless barley bread compared with 100% wheat bread. The beneficial effects on postprandial glucose metabolism of AmOn and hulless barley bread compared to 100% wheat bread may partly be due to a higher fibre content. Thus, previous studies have found that glycaemic responses are strongly related to β-glucan content and that addition of β-glucan reduces GI in subjects with either T2D [7–9, 24]. However, we cannot rule out that some hard to degrade amylose is accounted for as fibre when using the Megazyme total fibre assay kit. Thus, a high content of RS and fibres, has previously been observed to decrease GIP and GLP-1 with mixed results on subjective satiety [25–27]. We found that the GIP responses were reduced to 50% AmOn compared to all other three bread types and after 75% barley compared to 100% wheat being in line with previous studies in T2D where the acute GIP response was lower in T2D after a high fibre meal [23]. In contrast; however, we did not observe any differences in the postprandial response of GLP-1 to the four bread types.

The observed glycaemic responses to AmOn corresponded with in vitro studies, where AmOn bread showed lower predicted glycaemic responses than regular barley [15].

The reason for the lower blood glucose response to the genetic modified high-amylose barley product may be explained by various mechanisms. Amylose has extensive hydrogen bonds, is slow to gelatinise on cooking and therefore requires more energy to break [28]. High amylose content contributes to reduced gelatinization, impeding enzyme accessibility, which then reduces the rate of digestion, resulting in lowered postprandial glucose responses [29, 30]. High amylose starch will also render a hard shell on the surface of the starch granule, which increases the resistance of starch to digestion [31, 32]. The amount of resistant starch type 3 (RS3) formed under retrogradation is also much higher in the amylose-only starch type than in starch types with normal amylose content [14]. Apart from amylose content, other starch properties such as amount of RS, granule size, architecture, non-starch components as well as processing method are also considered to be important when assessing its impact on postprandial glucose responses [16, 31, 33–36].

It has also been found that the AmOn barley has a significant effect on composition and growth of gut microbiota in vitro [15]. It could have been interesting to investigate gut microbiota in our in vivo study as well; however, we presume that such an effect on the gut microbiota would need more time to develop than the present study period.

Our acute study does not add information on how mixing wheat flour with hulless barley or AmOn may influence weight or satiety. Previous human studies have reported mixed results on the effect on satiety after replacement of digestible starch with RS [26, 37]. Interestingly, previous animal studies showed that hulless barley compared to wheat elicited lower body weight gain and improved insulin sensitivity [38].

The baking quality of barley flour is less than that of wheat flour. Bread volume and pore size in the crumb is dependent of the strength and elasticity of the protein gluten network formed by interconnecting disulphide bonds in the dough [39]. This gluten network consists mostly of high molecular weight glutenin subunits (HMW-GS). In most wheat varieties the HMW-GSs are encoded by 4-5 different genes, whereas the barley genome only contains one gene homologous of the wheat HMW-GS, called D-hordein [40]. This reduction in the level and/or diversity of glutenin subunits directly affects gluten strength and bread volume. Barley breads are generally more compact and regarded less attractive to consumers than wheat breads. Therefore, there is a need for improving breeding techniques and the use of baking additives e.g., enzymes or hydrocolloids to increase bread volume, crumb pore size and sensory attractiveness of barley breads further.

The single-blinded, randomised, cross-over design is a strength of our study. However, it has the limitation that using AmOn is not commercially an option in Denmark due a prohibition of genetic modified breads here. Consequently, we are developing AmOn barley-varieties according to publicly accepted protocols. By improving baking quality of hulless barley and increasing the amylose content it may have a future potential as functional food for prevention and management of T2D.

In conclusion, the present study shows that consuming bread made by replacing wheat flour by either 50% AmOn or 75% hulless barley lowers postprandial glucose in subjects with T2D. However, long-term studies with bread made by replacement of wheat flour bread with hulless barley and AmOn flour bread as part of the diet are requested to clarify effects on both diabetes regulation, body weight, lipid metabolism, satiety and gut microbiome.

## Data Availability

Data from the study can be found within the article and additional data are available from the corresponding author on reasonable request.
